# Nanomedicine‐Enabled/Augmented Cell Pyroptosis for Efficient Tumor Nanotherapy

**DOI:** 10.1002/advs.202203583

**Published:** 2022-10-20

**Authors:** Zheng Zhang, Yajun Zhou, Shuangshuang Zhao, Li Ding, Baoding Chen, Yu Chen

**Affiliations:** ^1^ Department of Ultrasound Affiliated Hospital of Jiangsu University Zhenjiang 212000 P. R. China; ^2^ Department of Ultrasound The Fourth Affiliated Hospital Nanjing Medical University Nanjing 210029 P. R. China; ^3^ Tongji University School of Medicine Shanghai Tenth People's Hospital Tongji University Cancer Center Shanghai Engineering Research Center of Ultrasound Diagnosis and Treatment National Clinical Research Center of Interventional Medicine Shanghai 200072 P. R. China; ^4^ Materdicine Lab School of Life Sciences Shanghai University Shanghai 200444 P. R. China

**Keywords:** Gasdermin family, nanomedicine, programmed cell death, pyroptosis, tumor therapy

## Abstract

The terrible morbidity and mortality of malignant tumors urgently require innovative therapeutics, especially for apoptosis‐resistant tumors. Pyroptosis, a pro‐inflammatory form of programmed cell death (PCD), is featured with pore formation in plasma membrane, cell swelling with giant bubbles, and leakage of cytoplasmic pro‐inflammatory cytokines, which can remodel the tumor immune microenvironment by stimulating a “cold” tumor microenvironment to be an immunogenic “hot” tumor microenvironment, and consequently augment the therapeutic efficiency of malignant tumors. Benefiting from current advances in nanotechnology, nanomedicine is extensively applied to potentiate, enable, and augment pyroptosis for enhancing cancer‐therapeutic efficacy and specificity. This review provides a concentrated summary and discussion of the most recent progress achieved in this emerging field, highlighting the nanomedicine‐enabled/augmented specific pyroptosis strategy for favoring the construction of next‐generation nanomedicines to efficiently induce PCD. It is highly expected that the further clinical translation of nanomedicine can be accelerated by inducing pyroptotic cell death based on bioactive nanomedicines.

## Graphical abstract

Nanomedicine‐induced pyroptosis for efficient tumor therapeutics is increasingly prominent at current stage. This review provides a comprehensive summary and deep discussion on the very‐recent progress achieved in this emerging field.

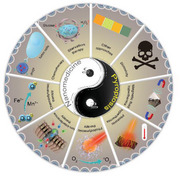

## 1 Introduction

Malignant cancer is severely threatening the health of human beings, and the incidence of all cancer cases is predicted to rise to 22.2 million worldwide by 2030.^[^ ^1^ ^]^ Refractory cancers can counter the tendency for apoptosis and achieve survival advantages through blocking proliferative apparatus during cancer proliferation and metastasis.^[^ ^2^ ^]^ Effective introduction of nonapoptotic programmed cell death (PCD) holds a promise as an alternative approach to rechallenge the anti‐apoptosis malignant tumors.^[^ ^3^ ^]^ The previous view of apoptosis as the predominant PCD underlying cancer pathogenesis and therapeutics has largely changed, because cancer cells can undergo diverse types of cell death, such as pyroptosis.^[^ ^4^ ^]^ Pyroptosis, the inflammasomes‐induced PCD, is induced by members of Gasdermin superfamily, including GSDMA, GSDMB, GSDMC, GSDMD, and GSDME (originally identified as DFNA5).^[^ ^5^ ^]^ Gasdermin proteins have been proved to have intrinsic cytotoxic Gasdermin‐N fragments, which are often covered by inhibitory Gasdermin‐C fragments.^[^ ^6^ ^]^ Proteolytic cleavage of the connective loop between Gasdermin‐C and ‐N fragments liberates the effective Gasdermin‐N terminals to translocate the plasma membrane.^[^ ^7^ ^]^ This pore‐forming activity undermines cellular osmotic potential, causing balloon bulge from the membrane and the leakage of inflammatory factors including interleukin (IL)‐1*β*, IL‐18, and high‐mobility group box 1 (HMGB1) into the extracellular environment to amplify the local and systemic inflammatory effects.^[^ ^8^ ^]^ Pyroptosis, first mistakenly outlined as apoptosis, was initially discovered as lysis happening following infection of bacteria or pathogens in myeloid cells as early as the 1990s,^[^ ^8^, ^9^ ^]^ which can be obviously distinguished from other PCD due to the unique morphological and biochemical characteristics.^[^ ^8i^ ^]^ In addition, several researches have been given notice that specific metal ions, small molecules and clinical chemotherapeutic drugs are capable of initialing Gasdermin family‐induced pyroptosis in a diverse variety of malignant cancers.^[^ ^10^ ^]^ However, there are several challenges in the administration of these pyroptosis‐inducers due to rapid clearance from systemic circulation, unsatisfactory biological distribution and inevitable side reactions. Moreover, increasing evidence has indicated that dysregulation of pyroptosis may decrease pathogen clearance, impair adaptive immune defense and affect every stage of carcinogenesis.^[^ ^11^ ^]^ Thus, the dual roles of pro‐cancer or pro‐“host” of pyroptosis compel the scientific community to make a terrific amount of efforts to seek out how to selectively utilize this double‐edged sword for efficient cancer therapy.^[^ ^12^ ^]^ Consequently, the exploitation and development of an efficient strategy to induce pyroptosis while minimizing nonspecific damage is highly urgent, necessary, and expected.

Nanotechnology has been changing the world all the time, which takes full advantage of the complicated theories, methodologies, and techniques developed in nanomedicine to provide unprecedented opportunities to change or even revolutionize our ways of lives.^[^ ^13^ ^]^ Especially, nanomaterials (materials at nanoscale size) have become a major research hotspot in today's scientific fields. Nanomaterials exhibit fascinating physicochemical properties with considerable biocompatibility, excellent tumor accumulation and real‐time feedback, endowing them with broad prospects in diverse application fields.^[^ ^14^ ^]^ For example, versatile bioactive nanomaterials combine diagnostic functions and therapeutic capabilities into a single nanoplatform with imaging capabilities such as computed tomography (CT), photoacoustic (PA) imaging, as well as magnetic resonance imaging (MRI), and therapeutic functions including photothermal therapy (PTT), photodynamic therapy (PDT), sonodynamic therapy (SDT), and chemodynamic therapy (CDT).^[^ ^15^ ^]^ More importantly, malignant cancers are generally characterized with quite intricate but unique tumor microenvironment (TME) such as mildly acidic condition, reducing environment, oxygen deficiency (hypoxia), and abundant intermediates,^[^ ^16^ ^]^ which either provides a suitable nutrition and environment for cancer proliferation and metastasis, or unlocks the “gate” for selective and efficient therapeutic modalities.^[^ ^17^ ^]^ Hence, nanomedicine‐enabled pyroptosis can not only make up for the defects and limitations of traditional therapeutic modality, but also improve the corresponding therapeutic efficacy and specificity.

**Figure 1:**
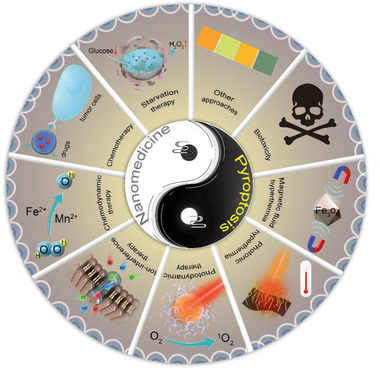
Summative scheme of nanomedicine‐enabled/augmented antitumor pyroptotic therapy.

Nanomedicine‐induced pyroptosis for efficient tumor therapeutics has been increasingly prominent at the current stage. In this review, we provide a comprehensive summary and deep discussion of the very recent progress achieved in this emerging field (**Table** **1**). First, bioactive nanoparticles can directly enable and promote pyroptosis for combating malignant tumors via photodynamic therapy (PDT), hyperthermia therapy (HT), chemodynamic therapy (CDT), starvation therapy, ion‐interference therapy (IIT), and chemotherapy. Second, the intrinsic biological toxicity of nanoparticles triggered by pyroptosis is also being paid more attention. Third, pyroptosis induced by other approaches and the potential mechanisms is also discussed in detail. Last but not the least, the critical issues on the current challenges and future developments of this emerging field are also emphatically discussed for potentiating their further clinical translations (**Figure** **1**). It is noted that the definite underling mechanisms of pyroptosis activated by nanomedicine as discussed in this review will extensively broaden the understanding of the relationship between cell pyroptosis and cancer‐inhibitive activity, and further provide intriguing molecular avenues toward personalized medicine in combating malignant cancer.

**Table 1:** Summary of nanomedicine‐enabled/augmented pyroptosis for tumor nanotherapy

Categories	Nanomaterials	Gasdermin	Tumor cells	Mechanism	Reference
Photodynamic therapy	TBD	GSDMD	4T1, HeLa and C6 cells	Improve the PDT efficiency by pyroptosis‐inducing antitumor immune	[21]
	CA‐Re	GSDMD	4T‐1 cells	PDT triggered immunogenic pyroptosis to enhance DCs maturation and systemic immune effects	[22]
Magnetic fluid hyperthermia	Gastrin‐MNPs		INR1G9‐CCK2R cells	Increase the temperature and promote cytotoxic free radical generation for pyroptosis	[23]
	TMZ/Fe‐TSL	GSDMD	U87 and U251 cells	Elevate the temperature in tumor site for inducing pyroptosis under the AMF irradiation	[24]
Photonic hyperthermia	BNP	GSDME	4T‐1 cells	Regulate immunosuppressed TME and improve the immune responses	[26]
Chemodynamic therapy	VTPA	GSDMD	4T‐1 cells	Intracellular release of Mn ions and iron oxide nanoparticles to produce abundant ROS through Fenton‐like reactions	[30]
	FeSO_4_	GSDME	A375	Iron‐induced CDT for oligomerization of Tom20 for pyroptosis	[31]
Starvation therapy	PICsomes		4T‐1 cells	Glucose depletion amplified the oxidative stress in cascade reaction to increase pyroptotic efficiency	[33]
Ion‐interference therapy	Lip‐MOF	GSDMD	HeLa cells	Increase the intracellular Fe ion concentration to trigger pyroptosis	[36]
	NaCl nanocrystals	GSDMD	HepG2 cells	GSH‐responsive explosive ion to induce pyroptosis	[39]
	CaNMs	GSDME	4T‐1 cells	Calcium overload‐inducing pyroptosis to improve DCs maturation and systemic immune effects	[43]
Chemotherapy	Lipo‐DDP	GSDME	4T‐1 cells	Improve the immune response of chemotherapy by epigenetic‐based pyroptosis	[46]
	As_2_O_3_‐NPs	GSDME	Huh7 cells	Contribute to improving the efficiency of As_2_O_3_‐induced pyroptosis	[48]
	PDNP	GSDME	CT26 and 4T‐1 cells	Chemotherapy‐inducing pyroptosis to increase the antitumor immune response	[49]
Multipronged therapies	MCCP	GSDME	4T‐1 cells	Pyroptosis induced by CDT and chemotherapy to boost anti‐PD‐1 efficiency for immune therapy	[50]
	OMVs	GSDMD	4T‐1 tumor	Combinational photodynamic/chemo‐/immunotherapy inducing pyroptosis	[51]
Biological toxicity	MSNs		L02 cells	NLRP3 inflammasome activation causing liver inflammatory response	[54]
	fumed SiO_2_	GSDMD	KUP5 cells	Intracellular K^+^ efflux for NLRP3 inflammasome activation responsible for liver damage	[56]
	CB		RAW264.7 cells	Amplify oxidative stress for inflammasome activation	[57]
	AgNP_15_		THP‐1 cells	ER stress caused by ATF‐6 degradation and NLRP‐3 inflammasome	[59]
	MOx	GSDMD	KUCs	Lysosomal damage and NLRP3 inflammasome activation	[61]
	ITO		THP‐1 cells	Inflammatory disorders induced by NLRP3‐ASC inflammasome in macrophages	[62]
Others	GSMDA‐NP	GSDMA	4T‐1 cells	Trigger proptosis to improve the efficacy of cancer immunotherapy	[65]
	ANPS	GSDME	A549 cells	Activate pyroptosis inside the early endosomes with minimum side effects	[69]
	*Lmo*@RBC	GSDMC	CT26 cells	Increase the antitumor immunity of *Lmo‐*induced pyroptosis	[72]

## 2 Application of Nanomedicine in Enabling Pyroptosis

### 2.1 Photodynamic Therapy‐Enabling Pyroptosis

**Figure 2:**
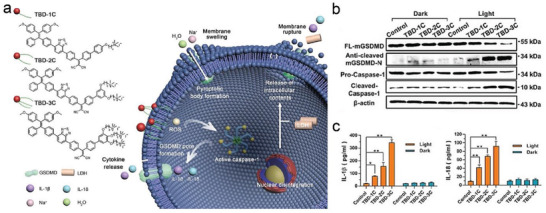
a) Illustration of the pyroptosis pathway activated by three AIE photosensitizers TBDs. b) Cleavage of GSDMD and Caspase‐1 were observed in response to PDT stimulation by western blot assay. c) IL‐1b and IL‐18 release into the supernatant was measured by ELISA in 4T‐1 cells after different treatments. Reproduced with permission.^[^ ^21^ ^]^ Copyright 2021, Wiley‐VCH.

Photodynamic therapy (PDT) is a clinically approved and useful therapeutic modality for several kinds of malignant tumors that has become an alternative strategy to conventional radio/chemotherapy.^[^ ^18^ ^]^ The advantages of PDT include spatiotemporal controllability, non‐invasiveness, and few adverse responses. At present, the underlying mechanism of PDT involves the transport of photosensitizers into the targeted tumor area, and then excitation with a specific wavelength of light to produce harmful reactive oxygen species (ROS) to initiate the photochemical response.^[^ ^19^ ^]^ Owing to the capability to release proinflammatory cytokines and potentiate the immunogenic effects, cell pyroptosis has significant potential as a unique cell death pathway that could play an adjunct part in photodynamic tumor therapy.^[^ ^20^ ^]^ For example, the prospective membrane anchoring aggregation‐induced emission (AIE) photosensitizers were designed for PDT‐triggered pyroptotic cell death.^[^ ^21^ ^]^ These AIE photosensitizers (TBD‐1C, TBD‐2C, and TBD‐3C) were synthesized to modulate the number of cationic chains attached to the aromatic structure (**Figure** **2**a). By applying the laser radiation, the produced ROS contributed to in situ membrane destruction and subsequent cellular lysis, thus resulting in efficient pyroptosis occurrence. Moreover, along with the increase of cationic chains, the membrane‐anchoring capability of AIE photosensitizers was dramatically improved, and the initiated cell pyroptosis gradually occupied the predominant cell death in photodynamic tumor therapy. In in vitro experiments, the levels of pyroptotic proteins (caspase‐1 and GSDMD), and the concentrations of proinflammatory cytokines (IL‐18 and IL‐1*β*) were both dramatically increased in the group of TBD‐R + light excitation, as measured by western blot and ELISA, respectively (Figure 2b,c). Light‐excited AIE photosensitizers can efficiently drive cell pyroptosis and then initiate a series of antitumor immune responses, which provides a distinct paradigm for broadening the application of PDT in tumor suppression.

**Figure 3:**
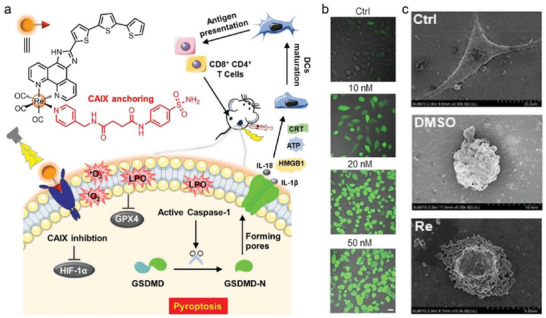
a) Structure of CAIX‐anchored photosensitizer (CA‐Re), which was able to induce membrane rupture, as well as drive cell pyroptosis and antitumor immunity under NIR irradiation. b) ROS generation in a concentration‐dependent behavior under NIR irradiation. c) SEM images of MDA‐MB‐231 cells after different treatments. Reproduced with permission.^[^ ^22^ ^]^ Copyright 2021, Wiley‐VCH.

In addition, Su et al. also developed a carbonic anhydrase IX (CAIX)‐anchored rhenium(I) photosensitizer (CA‐Re), which could not only carry out type‐I and type‐II PDT with high efficiency but also initiate GSDMD‐derived pyroptotic death to promote the immunogenicity (**Figure** **3**a).^[^ ^22^ ^]^ The ROS level in CA‐Re treated normoxic cells was negligible in the darkness, but ROS generation was significantly elevated in a concentration‐dependent manner under near infrared (NIR) irradiation (Figure 3b). As shown in SEM observation, the CA‐Re treating tumor cells exhibited pyroptotic features with fried egg cells with flat cytoplasm and intact nuclei located on the main plane, which was visibly different from the control cells and apoptotic cells treated by dimethyl sulfoxide (DMSO) (Figure 3c). The expressions of pyroptotic markers (caspase‐1 and GSDMD) and the levels of immune cells (DCs, CD4^+^, and CD8^+^) were both visibly increased in the CA‐Re under light irradiation, as detected by western blot and ELISA, respectively. PDT‐induced pyroptosis could improve tumor immunogenicity, and promote the adaptive immune responses to eliminate primary tumors at the same time as suppressing distant tumors. These emerging PDT‐induced pyroptosis strategies not only provide ideas for the design of more efficient photodynamic tumor treatments but also inspire us to explore other unknown therapeutic modalities for inducing pyroptosis.

### 2.2 Pyroptosis Promoted by Hyperthermia Therapy

#### 2.2.1 Magnetic Fluid Hyperthermia

**Figure 4:**
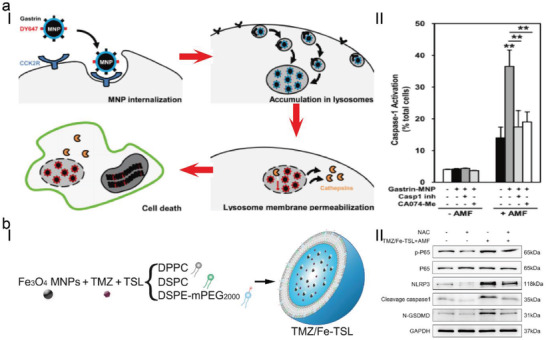
a) Schematic illustration of MFH‐triggered pyroptosis. Under the exposure of AMF, Gastrin‐MNPs resulted in lysosome membrane permeabilization, and the release of Cathepsin‐B into the cytoplasm, which eventually caused the induction of cell pyroptosis and Caspase‐1 activation of cells labeled with FAM‐FLICA‐Casp1. Reproduced with permission.^[^ ^23^ ^]^ Copyright 2018, Elsevier. b) Schematic representation of TMZ/Fe‐TSL and pyroptosis‐related protein expressions in U87 cells after different treatments. Reproduced with permission.^[^ ^24^ ^]^ Copyright 2022, Elsevier.

Magnetic fluid hyperthermia (MFH) involves the combination of magnetic nanoparticles (MNPs) with an alternating magnetic field (AFM), which generates local hyperthermia in tumors without the limitation of penetration depth. On this basis, the gastrin and NHSDY647‐PEG1 modified magnetic iron oxide nanoparticles (Gastrin‐MNPs) were developed to be specifically internalized by tumor cells and subsequently gathered into lysosomes to cause nonapoptotic cell death upon the AMF.^[^ ^23^ ^]^ The lysosome‐accumulated MNPs could increase the temperature very locally and promote cytotoxic free radical generation, which induced lipid peroxidation, lysosome membrane permeabilization, and leakage of lysosomal enzymes into the cytoplasm (**Figure** **4**a). More interestingly, the in vitro experiment was performed to demonstrate that cell death caused by MFH was not dependent on the caspase‐3 mediated apoptotic pathway, but was closely dependent on caspase‐1 and Cathepsin‐B induced pyroptotic pathway. Coincidentally, to enhance the tumor accumulation, the temozolomide and magnetic Fe_3_O_4_ were encapsulated by the temperature‐sensitive liposomes (TMZ/Fe‐TSL) to heat the local glioblastoma (GBM) cells to about 42 °C without damaging surrounding normal tissues in the presence of AMF (Figure 4b).^[^ ^24^ ^]^ The heating temperature promotes transient opening of the blood–brain barrier (BBB), facilitates the on‐demand drug release, and improves the generation of free radicals. The cell viability treated by TMZ/Fe‐TSL + AMF demonstrated the enhanced cell death rates in both U251 cells and U87 cells (56.13 ± 6.124% and 48.35 ± 0.495%). The pyroptosis‐related proteins (NLRP3, caspase‐1, and GSDMD‐N) were significantly upregulated compared to the control group, while the expression of apoptotic protein (caspase‐3) displayed a reverse change. These significant advances in the understanding of the underlying mechanism of cell pyroptosis caused by the MFH effect are helpful to promote the optimization of strategies from a therapeutic perspective.

#### 2.2.2 Photonic Hyperthermia

**Figure 5:**
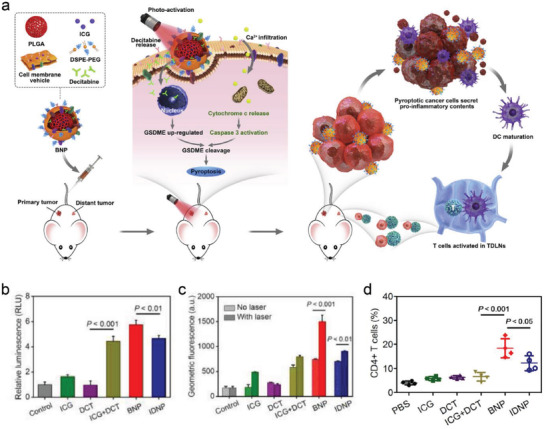
a) Schematic illustration of photoactivated pyroptosis for anti‐tumor immunotherapy. b–d) The level of GSDME N‐terminals, caspase‐3, and CD4^+^ T cells after different treatments, respectively. Reproduced with permission.^[^ ^26^ ^]^ Copyright 2020, Elsevier.

Photonic hyperthermia (PTT) triggers tumor ablation by absorbing light energy into hyperthermia with the help of photothermal agents and displays great clinical transform potentials.^[^ ^25^ ^]^ Certain nanoparticles have been extensively exploited as near‐infrared (NIR)‐assisted photothermal agents, and have also exhibited outstanding photothermal effects and satisfactory biosafety. It is noted that PTT has also shown its capability to generate pyroptotic antitumor immunological effects by reversibly disrupting cell membranes. For instance, a biomimetic nanoparticle (BNP) composed of a poly(lactic‐*co*‐glycolic acid) (PLGA) polymeric core and a cell membrane shell was developed and designed, which was used to combine photothermal effects to drive cell pyroptosis for promoting cancer immunotherapy (**Figure** **5**a).^[^ ^26^ ^]^ Cell membrane shell endowed BNP with tumor‐targeting capability and low immunogenicity, and meanwhile, photothermal agents like indocyanine green (ICG) could convert low‐dose light energy into a local hyperthermia effect within tumor tissues. Due to the tumor‐targeting capability and low immunogenicity, BNP was readily internalized in the tumor, and ICG punctured the plasma membrane to cause a remarkable intracellular Ca^2+^ concentration increase upon low‐dose NIR irradiation for hyperthermia generation. GSDME expression was dramatically decreased while GSDME‐N terminal and caspase‐3 cleavage were remarkably elevated upon photoactivation (Figure 5b,c). In particular, elevated percentage of CD4^+^ T cells in distant tumors was found after BNP + photoactivation (Figure 5d), indicating that this therapeutic modality was able to activate systematic T cells for antitumor immunotherapy. Given the substantial potential for antitumor, antimetastasis, and systemic T‐cell activation, the pyroptosis‐related BNP offers an emerging strategy for cancer immunotherapy with broad clinical feasibility and desirable biocompatibility. Shen et al. also designed and constructed a polydopamine (PDA)‐coated metal–organic framework (MOF) containing IR820 (MP@PI), which could generate excessive iron and hyperthermia after laser excitation, thereby eliciting the efficient pyroptosis and evoking the powerful immune response.^[^ ^27^ ^]^ Based on the nanomaterials‐enabled heat production via laser radiation, these results provided evidence for the capability of heat to induce pyroptosis and propel interest in constructing other PTT‐triggered pyroptosis nanosystems.

### 2.3 Pyroptosis Promoted by Chemodynamic Therapy

**Figure 6:**
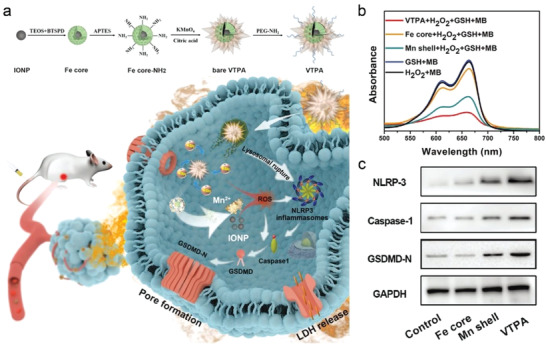
a) Schematic diagram of the synthetic process of spiky structure of VTPA and the mechanism of pyroptosis induced by VTPA on the basis of Fenton‐like reaction. b) MB degradation by Fenton and Fenton‐like reaction mediated by VTPA, Mn shell, and iron oxide core. c) Western blot analysis of NLRP3, Caspase‐1, and GSDMD‐N after various treatments. Reproduced with permission.^[^ ^30^ ^]^ Copyright 2021, Wiley‐VCH.

Chemodynamic therapy (CDT) has been proposed as a promising tumor intervention by triggering actual redox reactions for excessive production of highly toxic ROS based on Fenton or Fenton‐like reactions.^[^ ^13b^ ^]^ Driven by the recent advancement in nanochemistry, a lot of nanocatalysts have been applied to tumor sites to launch catalytic reactions to perform therapeutic effects. Based on the different biological metabolic pathways of cancer cells and normal cells, CDT applies exclusive biochemical states, such as mild acidity and excessive hydrogen peroxide (H_2_O_2_), to promote the Fenton reaction in tumors. Despite there being differentiation of the composition and structure of tumor microenvironment (TME) among various kinds of cancers, several common features still exist, including hypoxia, mild acidity, and overproduction of glutathione (GSH) and H_2_O_2_.^[^ ^28^ ^]^ Hence, the introduction of immobilized nanocatalysts into cancers to produce toxic species in situ may also be highly desirable for highly specific tumor treatments via Fenton and Fenton‐like reactions.^[^ ^29^ ^]^ Recently, a virus‐spike‐like tumor activatable pyroptotic agent (VTPA) was recently designed and synthesized, which was consisted of an organosilica‐coated iron oxide nanoparticles core and spiky manganese dioxide layer (**Figure** **6**a).^[^ ^30^ ^]^ VTPA could be degraded by the overexpressed GSH to release Mn ions and IONPs, and then produce abundant ROS through Fenton‐like reactions to amplify intracellular oxidative stress. To further verify the hydroxyl radicals (•OH) generation, the methylene blue (MB) absorbance was found to decrease noticeably in the presence of VTPA (Figure 6b). Furthermore, the cathepsin‐B released from the ruptured lysosomes and the high level of ROS synergistically activated the NLRP3 inflammasome, resulting in activating the cascade NLRP3/caspase‐1/GSDMD pyroptosis pathway (Figure 6c). The current study not only highlights the significance of nanostructure‐dependent lysosomal destruction, but also provides a Fenton‐like reaction for promoting ROS production and launching subsequent cell pyroptosis.

**Figure 7:**
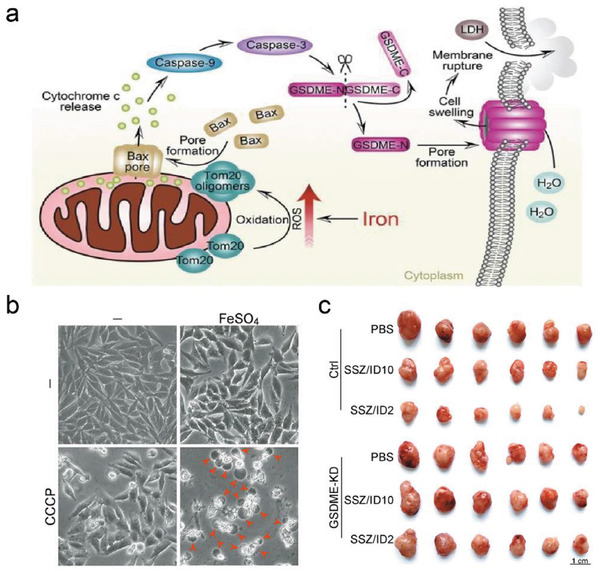
a) Schematic illustration of Tom20‐Bax‐caspase‐GSDME under CDT. b) The addition of FeSO_4_ to cause cell membrane bulges and pyroptosis induction (pyroptotic cells are shown by red arrows). c) Images of xenograft tumors from wild‐type and GSDME knockdown nude mice after different treatments. Reproduced with permission.^[^ ^31^ ^]^ Copyright 2018, Springer Nature.

In addition, Zhou et al. constructed FeSO_4_ NPs that could be rapidly ionized into Fe^2+^ in mildly acidic circumstances, and then catalyzed the reactant of H_2_O_2_ into the highly toxic •OH. This iron‐induced oxidative stress induced the oxidation and oligomerization of the mitochondrial outer membrane protein Tom20. Bax was brought into the mitochondria to aid in the release of cytochrome c for caspase‐9 and caspase‐3 activation, which subsequently led to pyroptotic death through the cleavage of GSDME (**Figure** **7**a).^[^ ^31^ ^]^ After CDT was monitored, cells resembled the typical pyroptotic cell shape with visible balloon‐like bubbles, whereas melanoma cells demonstrated the typical apoptotic morphology upon GSDME knockdown (Figure 7b). In vivo, A375 xenograft tumor model also indicated that such a combined CDT treatment favored improved cancer inhibition (Figure 7c). The above results prove that CDT‐induced pyroptosis features significant advantages in tumor suppression, which is especially beneficial for broadening the application scope of CDT‐involved tumor therapy.

### 2.4 Pyroptosis Facilitated by Starvation Therapy

**Figure 8:**
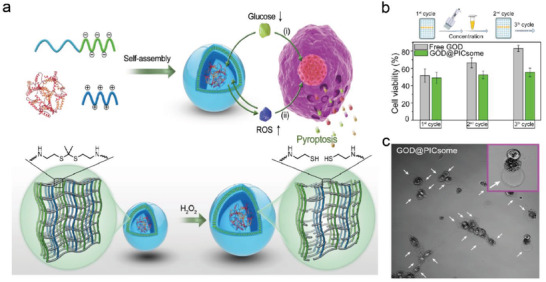
a) Schematic illustration of ROS‐responsive GOD‐loaded nanoreactor featuring self‐boosting catalytic glucose oxidation capability. b) Cytotoxicity of recycled GOD and GOD@PICsomes at their IC_50_ concentration. c) Bright field images of 4T‐1 tumor cells treated with GOD@PICsomes. Reproduced with permission.^[^ ^33^ ^]^ Copyright 2020, Wiley‐VCH.

Cancer cells commonly show stronger energy requirements than adjacent normal cells and absorb more lipids, amino acids and glucose from the surrounding microenvironment to guarantee sufficient substrates for ATP generation and metabolism. Thus, it is promising to realize tumor starvation therapy by destroying the nutrient influx of tumor cells. Currently, most nanomedicine‐based tumor starvation therapy focus on the manufacture of glucose oxidase (GOx)‐loaded nanosystems to catalyze glucose and O_2_ to generate gluconic acid and H_2_O_2_ to drive tumor starvation effects.^[^ ^32^ ^]^ On this ground, Li et al. synthesized a GOx‐loaded and ROS‐responsive therapeutic nanomaterial with self‐improving catalytic glucose oxidation and pyroptosis function based on polyion complex vesicles (PICsomes) (**Figure** **8**a).^[^ ^33^ ^]^ The increase in vesicle volume resulted in a significant decrease in membrane cross‐linking density and an obvious increase in membrane permeability, which was beneficial for increasing the efficacy of catalyzing glucose oxidation. Despite the IC_50_ value of PICsomes being higher than free GOD treatment, GOD‐loaded PICsomes could ensure GOD to maintain long‐lasting cytotoxicity (Figure 8b). Moreover, the unique pyroptotic morphology with large bubbles, further validated that the cell pyroptosis occurred in tumor cells after the interference of PICsomes (Figure 8c). Nonetheless, several critical issues still remain to be dealt with in future research. On the one hand, TME inhibits the supplement of H_2_O_2_ under hypoxia conditions, because oxygen is necessary for glucose consumption. On the other hand, the GOD enzyme may react with glucose in normal tissues due to a lack of targeting to the TME of tumor cells, thus diminishing the selectivity of the reaction. As a result, more efforts and detailed researches should be devoted to this field of tumor starvation therapy‐enabled pyroptosis pathway.

### 2.5 Pyroptosis Mediated by Ion‐Interference Therapy

**Figure 9:**
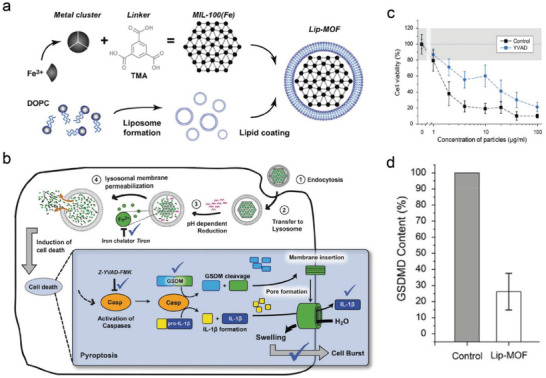
a) Schematic illustration of synthetic process of Lip‐MOF nanoparticles. b) The phagocytosis of Lip‐MOF nanoparticles resulting in cell pyroptosis in acidic microenvironments. c) MTT cytotoxic assay of tumor cells after varied treatments. d) GSDMD expression in tumor cells after varied treatments. Reproduced with permission.^[^ ^36^ ^]^ Copyright 2020, Wiley‐VCH.

Bioactive ions generated/released from nanomaterials participate in multiple physiological processes, including osmotic pressure and pH homeostasis, signaling cascade activation, enzyme activity, and biomolecule targeting.^[^ ^34^ ^]^ Their abnormal accumulation/distribution in cells can also interfere with normal physiological processes, such as irreversible biological destruction and activation of biochemical reactions with cytotoxic components’ production, subsequently inducing distinct kinds of programmed cell death.^[^ ^35^ ^]^ Therefore, ions can be exploited against a wide spectrum of cancers with high efficiency and without drug resistance, namely, ion‐interference therapy (IIT). Inspired by this, the liposome‐encapsulated MOFs (Lip‐MOF) were designed as pyroptotic inducers consisting of 1,2‐dileoyl‐snglycero‐3‐phosphocholine (DOPC) loading Fe^3+^ and trimesic acid‐based MOFs (**Figure** **9**a).^[^ ^36^ ^]^ The Lip‐MOF was readily endocytosed by cancer cells and released Fe^3+^ with the help of acidic conditions, which subsequently activated the caspase to cleave GSDMD into GSDMD‐N fragments for cancer cell pyroptosis (Figure 9b). After the addition of z‐YVAD‐FMK (an inflammatory caspase inhibitor), the cytotoxicity and GSDMD expression induced by Lip‐MOF were quietly reduced, suggesting that the cell pyroptosis was closely dependent on a caspase‐mediated cell death pathway (Figure 9c,d). This Lip‐MOF nanosystem represents a classical paradigm for tumor therapy via an ion interference‐mediated pyroptotic pathway.

**Figure 10:**
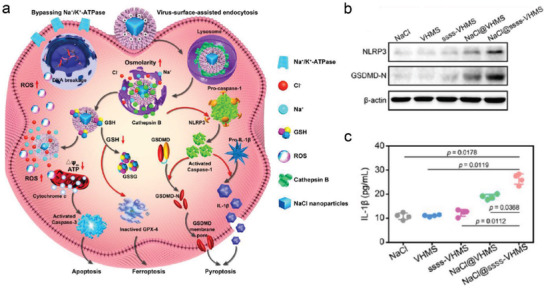
a) Illustration of tumor microenvironment‐responsive yolk–shell NaCl nanocrystal@virus‐inspired mesoporous tetrasulfide‐organosilica for synergistic ion interference therapy via caspase‐1‐dependent pyroptosis. b) Western blot analysis of NLRP3 and GSDMD‐N terminals expression after various treatments. c) ELISA assay kit of IL‐1*β* secretion after various treatments. Reproduced with permission.^[^ ^39^ ^]^ Copyright 2022, American Chemical Society.

Moreover, cells maintain low ratios of inside to outside Na ions to sustain the extracellular/intracellular pH balance, protein transport, and cellular volume regulation.^[^ ^37^ ^]^ It is well‐known that introducing cells into a hyperosmotic condition to reduce the extracellular osmolarity of Na ions can give rise to cellular swelling, membrane cytoskeleton destruction, and even cell death.^[^ ^35c^ ^]^ Therefore, a similar phenomenon can be produced through the delivery of excessive Na ions into tumor cells to elevate intracellular osmolarity.^[^ ^38^ ^]^ Li et al. first designed and synthesized GSH‐responsive virus‐inspired hollow mesoporous tetrasulfide‐organosilica‐modified NaCl nanocrystals (NaCl@ssss‐VHMS) for in situ gradual degradation into Na^+^/Cl^−^ and elevating the intracellular osmolarity, which was not dependent on the transport protein.^[^ ^39^ ^]^ The damage to endo/lysosomes caused by the increase of intracellular osmolarity could then cause cathepsin B release, activating the NLRP3 inflammasome, and triggering caspase‐1 dependent pyroptosis (**Figure** **10**a). In addition, the NaCl@ssss‐VHMS could also remarkably exhaust intracellular over‐expressive GSH to weaken the self‐defense of tumor cells, subsequently triggering GPX4‐dependent ferroptosis. Consequently, the synergy of ROS and osmotic pressure‐triggered cell pyroptosis effectively suppressed tumor growth. The results of western blot and ELISA assays revealed that the expression of NLRP3, GSDMD‐N terminals, and the cytokine IL‐1 were significantly increased, indicating that NaCl@sss‐VHMS is required for the occurrence of pyroptosis (Figure 10b,c).

**Figure 11:**
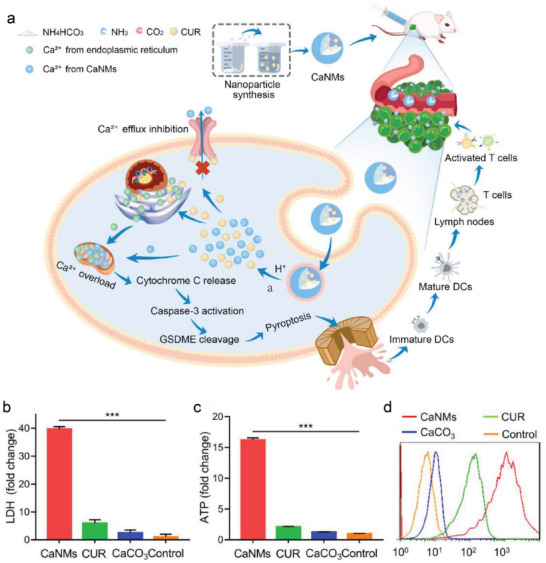
a) Schematic diagram of biodegradable Ca^2+^ nanomodulators as the pyroptosis inducers to activate pyroptosis through mitochondrial Ca^2+^ overload. b) The release of LDH, c) the release of ATP, and d) CRT expressions after different treatments. Reproduced with permission.^[^ ^43^ ^]^ Copyright 2022, Wiley‐VCH.

Calcium ion (Ca^2+^), as a ubiquitous second messenger at the cellular function, is related to multiple biochemical signal transduction pathways and also participates in the modulation of critical physiological activities.^[^ ^40^ ^]^ Recently, a distinct Ca^2+^‐based antitumor nanotherapy was developed, in which CaO_2_‐induced intracellular Ca^2+^ overload and exogenous ROS elevation were demonstrated.^[^ ^41^ ^]^ The triggered calcium overload was not only a trivial process, but also could be an effective “destructive factor” against malignant tumors, which has been defined as “calcicoptosis.”^[^ ^42^ ^]^ Inspired by this, biodegradable Ca^2+^ nanomodulators (CaNMs) were designed as pyroptosis inducers via mitochondrial Ca^2+^ overload.^[^ ^43^ ^]^ CaNMs were composed of CaCO_3_ and curcumin (CUR). The combination of CaCO_3_ and CUR generated the effective Ca^2+^ nanomodulators to release Ca^2+^ directly in the cells, and then eventually accumulated in mitochondria, resulting in the mitochondrial Ca^2+^ overload (**Figure** **11**a). A sudden surge in Ca^2+^ ions quickly broke the dynamic balance of Ca^2+^ and caused mitochondrial Ca^2+^ overload, eventually resulting in an increase of ROS and the release of cyt *c*. In particular, the cyt *c* further activated caspase‐3 to cleave GSDME into cytotoxic GSDME‐N fragments, thus contributing to the occurrence of pyroptosis. The rupture of the plasma membrane could release inflammatory contents, such as lactate dehydrogenase (LDH), adenosine triphosphate (ATP), and calreticulin (CRT) (Figure 11b–d), which activated the adaptive immune response for tumor immunotherapy. This paradigm confirms that Ca^2+^ nanomodulators are effective as pyroptosis inducers for tumor immunotherapy through mitochondrial Ca^2+^ overload, which provides the distinct inspirations and strategies for IIT‐mediated pyroptotic treatments.

### 2.6 Pyroptosis Initiated by Chemotherapy

**Figure 12:**
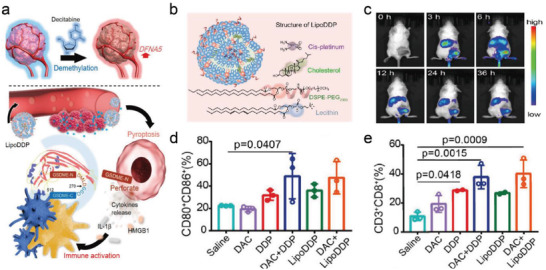
a) Illustration of DAC demethylation and immune activation triggered by Lipo‐DDP induced pyroptosis. b) A schematic illustration of lipo‐DDP synthesis process. c) Living fluorescence imaging of 4T‐1 tumor‐bearing mice after intravenous injection. d) Representative flow cytometric analysis of CD80^+^ and CD86^+^ cells within TDLN. e) Representative flow cytometric analysis of CD8^+^ and CD3^+^ T cells within the tumors. Reproduced with permission.^[^ ^46^ ^]^ Copyright 2019, American Chemical Society.

As the most common front‐line method in the clinic, chemotherapy plays a highly critical part in suppressing tumor proliferation and also prolonging the lives of tumor patients. However, chemotherapy‐caused drug resistance and unavoidably side effects are also practical trouble that should be resolved at the same time.^[^ ^44^ ^]^ Advanced nanotechnology could achieve chemotherapy by delivering chemotherapeutic agents in a more efficient and safer manner. Several chemotherapeutic drugs initiate caspase‐3‐induced apoptosis to kill cancer cells, which also lays the foundation for mediating the pyroptosis process based on GSDME (originally translated by DFNA5).^[^ ^45^ ^]^ However, most cancer cells lack the critical protein GSDME for caspase‐3‐derived cell pyroptosis, which is attributed to DFNA5 gene hypermethylation. In response to address this problem, a liposome‐based therapeutic strategy in which DNA methyltransferase decitabine (DAC) in combination with chemotherapy drugs cisplatin (Lipo‐DDP) was designed to induce the cascade pyroptosis in tumor site and activate the systemic immune responses via caspase‐3‐mediated pyroptosis pathway (**Figure** **12**a,b).^[^ ^46^ ^]^ Moreover, fluorescent live imaging was utilized to observe the body distribution and tumor accumulation of such a liposome‐based nanosystem. Benefiting from the efficient passive accumulation of enhanced permeability and retention (EPR) effect,^[^ ^47^ ^]^ Lipo‐DDP began to accumulate at the tumor tissue after 3 h intravenous injection, and of note, the fluorescence signal at the tumor site continued 36 h post‐injection, which guaranteed the basic therapeutic doses to trigger subsequent pyroptosis (Figure 12c). In vivo experiments demonstrated that the collaborative treatment of DAC and Lipo‐DDP largely boosted the enhancement of dendritic cells (DCs) maturation and cytotoxic T lymphocytes (CTLs) within the tumor microenvironment, indicating this combined treatment could be efficient for activating the immunological effect of the living system. (Figure 12d,e).

**Figure 13:**
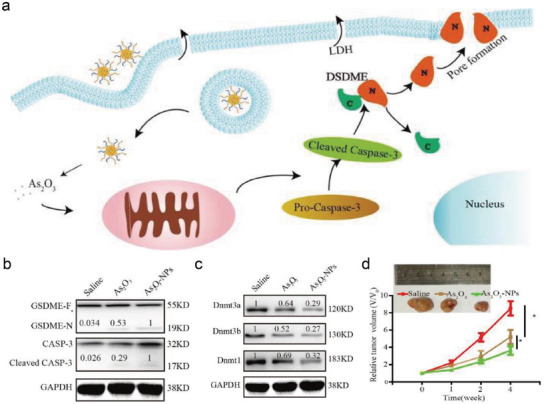
a) Schematic mechanisms of pyroptosis induced by As_2_O_3_‐NPs in tumor cells. b) The expression of GSDME‐F, GSDME‐N, caspase‐3, and cleaved caspase‐3 after various treatments. c) The expression levels of Dnmt3a, Dnmt3b, and Dnmt1 after different treatments. d) Changes in relative tumor volume of xenograft tumors during the therapeutic period. Reproduced with permission.^[^ ^48^ ^]^ Copyright 2019, Springer Nature.

To maintain the therapeutic As_2_O_3_ concentration at the tumor site while minimizing adverse side effects, Duan et al. designed and constructed a nanodelivery system in which the triblock copolymer monomethoxy (polyethylene glycol)‐poly(d, l‐lactide‐*co*‐glycolide)‐poly(l‐lysine) (mPEG‐PLGA‐PLL) was used to encapsulate chemotherapeutic drug As_2_O_3_ (As_2_O_3_‐NPs).^[^ ^48^ ^]^ Based on the mPEG‐PLGA‐PLL protection, As_2_O_3_‐NPs was equipped with several merits, including high stability, long systemic circulation, and efficient tumor accumulation. After As_2_O_3_‐NPs were phagocytosed by tumor cells, As_2_O_3_ was released to promote the activation of caspase‐3. Then GSDME was cleaved into cytotoxic GSDME‐N fragments to perforate the cell membrane, ultimately resulting in pyroptosis‐based cell death (**Figure** **13**a). In vitro results exhibited that As_2_O_3_‐NPs could either enhance the GSDME cleavage into the GSDME‐N terminals (Figure 13b) or downregulate DNA methyltransferase expression (Figure 13c). Moreover, in vivo experiments also demonstrated that As_2_O_3_‐NPs considerably reduced tumor growth, while free As_2_O_3_ exhibited moderate tumor inhibition, which was resulted from the protection and delivery of triblock copolymers (Figure 13d).

**Figure 14:**
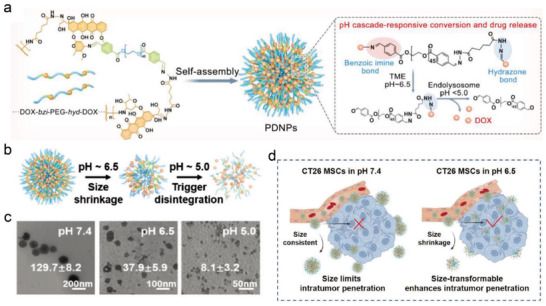
a) Chemical structure and synthesis of PDNPs and the potential mechanism of pH‐responsive sequential conversion. b) Schematic illustration of pH‐triggered cascade conversion of PDNPs. c) TEM images of PDNPs at different pH environments. d) Schematic illustration of the acid‐responsive size‐conversion PDNPs in CT26 MCSs. Reproduced under the terms of the Creative Commons CC‐BY license.^[^ ^49^ ^]^ Copyright 2022, The Authors. Published Wiley‐VCH.

Subsequently, a drug–polymer hybrid supramolecular nanoprodrug (PDNP) has been recently proposed as a pyroptosis inducer to augment immune responses.^[^ ^49^ ^]^ PDNPs were designed and constructed by several drug–polymer repeating units consisting of PEGylated DOX blocks, which were connected by two different acid‐cleavable linkers in response to the varying TME acidities (**Figure** **14**a). The PDNPs retained the “stealth” property of PEG at a physiological pH environment. Once reaching the acidic TME, PDNPs began to detach from PEG shielding and then immediately divided into small particles (Figure 14b,c). The subsequent size shrinkage happened after endocytosis, because the cleavage of the hydrazone bond was in response to the more acidic endolysosome, contributing to thorough PDNPs decomposition and release of chemotherapeutic drug DOX into the nucleus (Figure 14d). Thus, efficient penetration and accurate release of drugs in the tumor site could effectively drive GSDME‐initiated pyroptosis occurrence, which facilitates DC maturation, promoted CTL infiltration, and boosted the antitumor immune response. Because the role of chemotherapy‐triggered apoptotic death is commonly limited by the inherent or evolved resistance to apoptosis, the pyroptosis induced by chemotherapy offers a promising strategy to obviously increase the efficacy of anticancer therapy. This pyroptosis‐based chemotherapy dramatically improved tumor inhibition efficacy and also provided remarkable insights into tumor immunotherapy.

### 2.7 Pyroptosis Augmented by Multipronged Approaches

**Figure 15:**
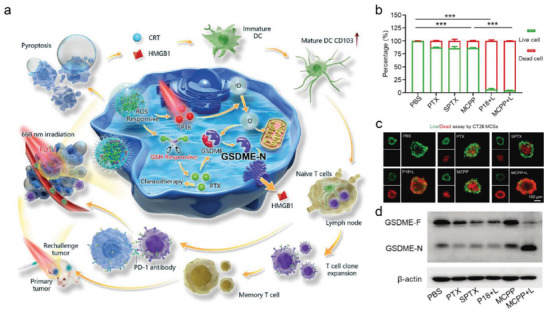
a) Schematic representation of the chemo‐photodynamic therapy‐activated pyroptosis for boosting immune checkpoint therapy. b) Quantitative analysis of live/dead experiments of CT26 cells after different treatments. c) CLSM image of the live/dead experiments of CT26 after different treatments. d) Western blot of GSDME and GSDME‐N terminals after various treatments. Reproduced under the terms of the Creative Commons CC‐BY license.^[^ ^50^ ^]^ Copyright 2021, The Authors. Published by Wiley‐VCH.

Pyroptosis has also been induced by multipronged nanotherapeutics. For instance, an innovative ROS/GSH dual‐responsive nanoplatform with paclitaxel (PTX) and photosensitizer purpurin 18 (P18) (MCPP) was constructed for chemo‐photodynamic tumor ablation to activate pyroptotic cell death (**Figure** **15**a).^[^ ^50^ ^]^ In this research, ROS generated by PDT not only achieved controlled nano‐prodrug release, but also induced efficient cell pyroptosis with PTX. The results of cell viability and live/dead experiments demonstrated the cytotoxicity of MCPP under laser irradiation was significantly enhanced than other groups, suggesting the highest tumor suppressive efficacy of the chemo‐photodynamic group (Figures 15b and 11c). The pyroptotic markers of caspase‐3 and GSDME‐N terminals were also remarkably increased in the MCPP with laser irradiation, as measured by west blot technology (Figure 15d).

Subsequently, Xiang et al. co‐loaded a PDT agent Ce6 and DOX into bacterial outer membrane vesicles (OMVs) for a combinational photodynamic/chemo/immunotherapy to induce pyroptosis occurrence.^[^ ^51^ ^]^ Ce6 and DOX were applied to induce PDT and chemotherapy, respectively. OMVs could recruit nonspecifically activated immune cells to initiate the antitumor immunotherapy. After laser irradiation, the pyroptosis‐related marker proteins, including NLRP3, cleaved caspase‐1, and cleaved GSDMD were significantly increased in both in vitro and in vivo experiments. Furthermore, pyroptosis‐related mRNAs (NLRP3, IL1*β*, caspase‐4, and NLRP12) were also dramatically elevated in transcriptomic analysis. Collectively, the obtained OMVs‐based nanosystem may shift tumor‐related macrophage M2‐to‐M1 polarization and activate pyroptosis‐related pathways to activate antitumor immune responses.

## 3 Intrinsic Biological Effect of Nanomaterials for Inducing Pyroptosis

Although ingenious chemical modifications including PEGylation are generally adopted to assist the nanomaterials to reduce their intrinsic biotoxicity, the biosafety impacts of these nanomaterials caused by pyroptosis have also raised great concern, especially in digestive and respiratory systems.^[^ ^52^ ^]^ In this section, we focused on the potential biotoxicity of bioactive nanomaterials caused by pyroptosis in the composition of nonmetal, and metal or metal oxide nanomaterials.

### 3.1 Nonmetal Nanomaterials

**Figure 16:**
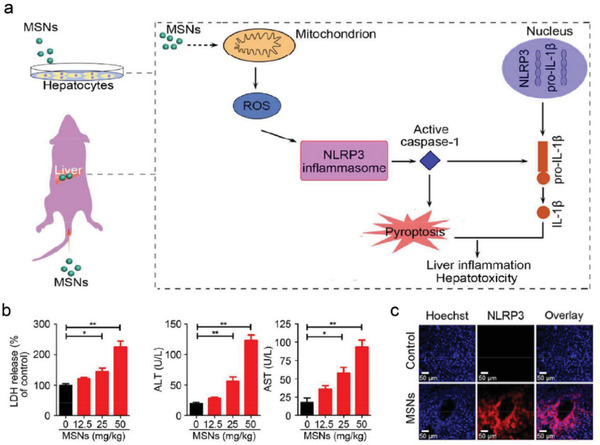
a) Schematic illustration of MSNs‐mediated pyroptosis and the related molecular mechanisms. b) The release of LDH, ALT and AST after different treatments. c) Immunofluorescence staining with anti‐NLRP3 antibody after MSNs treatments. Reproduced with permission.^[^ ^54^ ^]^ Copyright 2018, Royal Society of Chemistry.

Nonmetal nanomaterials have been extensively used in manufacturing industries, such as cosmetics, food, coatings, plastics, and electronic products.^[^ ^53^ ^]^ Due to their easy exposure to human beings and the surrounding environment, the security of nonmetal nanomaterials has attracted broad concern in the scientific community. It is noted that multiple pro‐inflammatory cytokines leakage during pyroptosis usually leaves normal tissues/cells in a chronic inflammatory environment, which inevitably causes adverse health effects in vulnerable human lives. For example, the biotoxicities of ultrasmall mesoporous silica nanoparticles (MSNs) were revealed to elicit the NLRP‐3 inflammasome activation in hepatocytes, leading to caspase‐1‐dependent pyroptosis in normal liver tissues (**Figure** **16**a).^[^ ^54^ ^]^ A dose‐dependent increase in lactate dehydrogenase (LDH), alanine aminotransferase (ALT), and aspartate aminotransferase (AST) was found in MSNs‐treated hepatocytes, indicating that MSNs caused obvious hepatotoxicity in hepatocytes (Figure 16b). In vivo assays illustrated that the NLRP3 inflammasome was absolutely responsible for pyroptosis after the administration of MSNs (Figure 16c). Thus, appropriate surface chemical modification of MSNs is helpful to reduce the liver enrichment and then improve the biocompatibility of MSNs.

**Figure 17:**
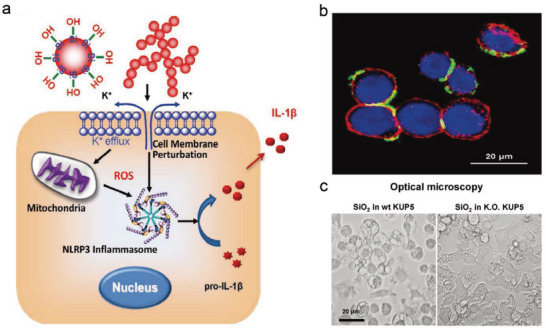
a) Schematic diagram of the fumed SiO_2_‐NPs inducing pyroptosis, accompanied by plasma membrane perturbation, potassium efflux, and NLRP3 inflammasome activation. b) SiO_2_‐NPs‐triggered surface membrane perturbation. Confocal microscopy images of KUP5 cells treated with FITC‐labeled fumed SiO_2_‐NPs. c) Optical microscope images to compare the morphological changes by fumed SiO_2_‐NPs in wild‐type and GSDMD^−/−^ KUP5 cells. Reproduced with permission.^[^ ^56^ ^]^ Copyright 2020, Wiley‐VCH.

In addition, SiO_2_ exposure was evaluated to be ≈100 mg every day. Feeding of food‐grade fumed silica (fumed SiO_2_) could also cause liver fibrosis in rats.^[^ ^55^ ^]^ However, there is relatively little information about the effects of silica nanoparticles on Kupffer (KUP5) cells compared to their effects on hepatocytes. Wang et al. found that fumed SiO_2_ could trigger K^+^ efflux in KUP5 cells and play a responsible role in lytic cell death (**Figure** **17**a).^[^ ^56^ ^]^ Confocal microscopy images showed that robust fumed SiO_2_ was closely adhered to the cell surface membrane (Figure 17b) and the accompanying surface membrane perturbation could efficiently trigger K^+^ efflux, NLRP3 inflammasome activation and cleavage of GSDMD into cytotoxic GSDMD‐N fragments for executing pyroptosis. The role of GSDMD as a critical protein in fume SiO_2_‐induced pyroptosis was confirmed by siRNA knockdown of GSDMD in KUP5 cells (Figure 17c). This research provides original insights into the liver damage from pyroptosis caused by silica, and further promotes the potential approach to enhance the security evaluation of nonmetal nanomaterials.

**Figure 18:**
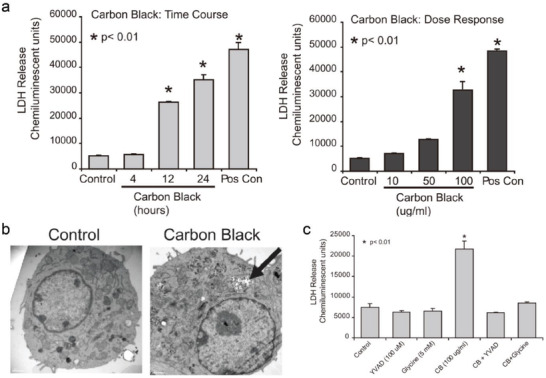
a) LDH release from RAW164.7 cells exposed to CB nanoparticles at varied times and concentrations. b) TEM images of control and CB nanoparticles exposed in the cellular region. c) LDH release of RAW164.7 cells after various treatments. Reproduced with permission.^[^ ^57^ ^]^ Copyright 2011, Elsevier.

Furthermore, inhalation of ultrasmall nanoparticles could bypass the mucociliary clearance and penetrate into the deeper sites of the respiratory tract. As a major component of environmental pollution and printer toner, carbon black (CB) nanoparticles are key targets for biotoxic evaluation. Inhalation of CB nanoparticles could efficiently trigger the release of LDH from macrophage RAW264.7 cells, indicating the destruction of the plasma membrane and subsequent cell pyroptosis induction (**Figure** **18**a).^[^ ^57^ ^]^ Transmission electron microscopy (TEM) was used to confirm CB exposed to macrophage regions (Figure 18b). To further investigate the underlying mechanism of cell death, the leakage of LDH was dramatically attenuated after the addition of YVAD (caspase‐1 inhibitor) and glycine (pyroptosis inhibitor) (Figure 18c), determining the cell pyroptosis caused by CD nanoparticles in RAW264.7 cells. Due to their involvement in ambient pollution and diesel exhaust, CB nanoparticles require more research to appropriately regulate their potential health hazards.

### 3.2 Metal and Metal Oxide Nanoparticles

Silver ion (Ag^+^) is a recognized star ion in the field of biomedicine that has been proven with excellent antimicrobial ability and extensively applied in remedying wounds, catheter and burns‐associated infections.^[^ ^58^ ^]^ These are broadly used in biomedical applications, and meanwhile increase the possibility of environmental exposure to silver nanomaterials. However, the nanosafety and biocompatibility of silver nanoparticles (Ag‐NPs) have not been thoroughly studied, so it is vital to clarify the exact mechanism of cell death in nanotoxicity evaluation at their molecular mechanism. Endoplasmic reticulum (ER) stress gives rise to an unfolded protein response, which is the main indicator of cytotoxicity. So far, activated transcription factor 6 (ATF‐6) has been performed as an ER stress sensor, and ATF‐6 cleavage produces cytoplasmic proteins as active transcription factors. On this ground, Girard et al. reported that AgNP_15_, depending on the concentration, could induce the rapid ER stress response with degradation of the ATF‐6, and then cause pyroptosis‐based cell death through NLRP‐3 inflammasome activation.^[^ ^59^ ^]^ Further research should be conducted to reduce the undesired underlying biotoxicity of Ag‐NPs for further promoting and strengthening their clinical translations.

**Figure 19:**
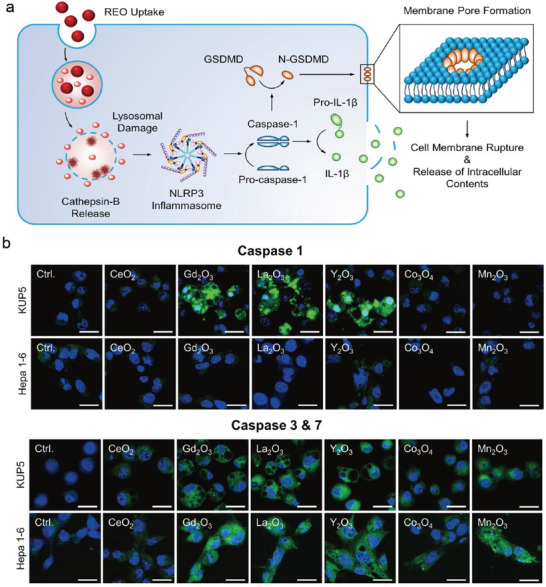
a) Schematic diagram showing the signaling pathway by which REOs caused pyroptosis pathway. b) Confocal microscopy images of the expression of caspase‐1, caspases‐3&7 in KUP5 and Hepa 1–6 cells after the treatments of various metal oxides. Reproduced with permission.^[^ ^61^ ^]^ Copyright 2018, American Chemical Society.

Apart from metal nanomaterials are extensively used in biomedicine, metal oxide (MOx) nanoparticles are the abundant engineered nanomaterials for diagnostic and therapeutic applications.^[^ ^60^ ^]^ Previous studies have demonstrated that after MO_X_ nanoparticles were deposited in the lungs and gastrointestinal tract, these nanoparticles could transfer within blood circulation, and then transport into the secondary organs to cause severe adverse effects.^[^ ^52a,b^ ^]^ As a secondary exposure site, the liver has been demonstrated to accumulate more MOx nanoparticles at much higher quantities compared with other organs. Hence, it is essential to conduct a biosafety screen to evaluate the interactions of MOx with phagocytic cells and hepatocytes. Mirshafiee et al. carried out a comparative analysis of the toxicological profiling of MOx nanoparticles in Kupffer cells (KCs), macrophages, and hepatocytes.^[^ ^61^ ^]^ Different toxicological profiles of rare‐earth oxides (REOs) and transition metal oxides (TMOs) were found in KCs, macrophages and hepatocytes, indicating different cell uptake processes of MOx could result in various cell death pathways. Among them, REOs (except CeO_2_) nanoparticles could trigger pyroptosis in KCs and other macrophage cell lines, which was apparently diminished after GSDMD knockdown (**Figure** **19**a). Further studies confirmed that this occurrence of pyroptotic death caused by REOs was premised on caspase‐1 and caspases‐3/7 in KUP5, rather than Hepa 1–6 cells (Figure 19b). Cellular responses of REOs were subsequently investigated in other cell types, including BMDMs, RAW 264.7, and J774A.1 cells, where it was shown that pyroptosis was a unique feature in phagocytic cells, but not in primary hepatocytes. This study not only focuses on the hepatotoxicity of MOx nanoparticles and the potential mechanisms at cellular/subcellular levels, but also offers a reference grid for biotoxicity screening of MOx nanoparticles in the liver organ.

**Figure 20:**
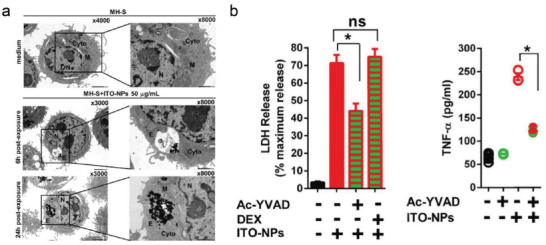
a) Evaluation of ITO nanoparticles on MH‐S cells. b) LDH and TNF‐*α* release behavior from MH‐S cells after different treatments. Reproduced with permission.^[^ ^62^ ^]^ Copyright 2016, Springer Nature.

The biotoxicity assessments of indium‐tin‐oxide (ITO) are worthy of concern because of the alveolar proteinosis and interstitial lung disease developed in workers who are in close contact with indium compounds. Nevertheless, the oncogenesis of these diseases is largely unknown. A study reported that intraperitoneally inoculated with ITO nanoparticles in mice could cause NLRP3‐dependent peritoneal inflammation, accompanied by neutrophil recruitment and IL‐1*β* release. Meanwhile, another research found the size of endosomes in MH‐S cells containing ITO‐NPs was closely in a time/concentration‐dependent manner (**Figure** **20**a).^[^ ^62^ ^]^ In vitro experiments showed that alveolar macrophages exposed to ITO‐NPs exhibited effective endocytosis of ITO nanoparticles and the release of tumor necrosis factor‐*α* (TNF‐*α*) and pyroptotic marker LDH, followed by cell lysis and cell death, which confirmed that ITO nanoparticles‐induced cell death was neither apoptosis nor necroptosis, but was caspase‐1‐dependent pyroptosis in MH‐S cells (Figure 20b). These researches performed the toxicological analysis of metal nanomaterials in biomedicine, which is helpful to provide useful information for the safety assessment and risk decision‐making  of these metal nanomaterials. Research in this area has a long way to go.

## 4 Pyroptosis Induced by Other Approaches

**Figure 21:**
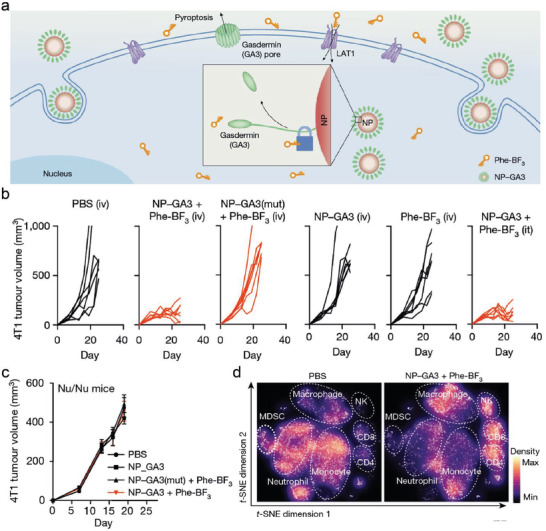
a) Schematic illustration of NP‐GSDMA3 for inducing pyroptotic therapy. b) Tumor volume curves in 4T‐1 tumor‐bearing mice as treated with different groups. c) Tumor volume curves in Nu/Nu mice bearing 4T‐1 tumors after varied interferences. d) Single‐cell RNA sequencing of immune cells isolated from 4T‐1 tumors with different treatments. Reproduced with permission.^[^ ^65^ ^]^ Copyright 2020, Springer Nature.

The expression of GSDM‐proteins is downregulated in different cancer cells, and these proteins are unraveled to possess antitumor pyroptosis properties.^[^ ^63^ ^]^ Consequently, the upregulation of GSDM‐proteins expression could activate cascade pyroptosis in tumor cells.^[^ ^64^ ^]^ The purified GSDM‐A3 protein conjugated to gold nanoparticles (GSDMA‐NP) was designed and developed via the triethylsilyl ether linkage.^[^ ^65^ ^]^ The phenylalanine trifluoroborate (Phe‐BF3) was introduced to cleave the ether linkage for the liberation of GSDM‐A3 from the designed GSDMA‐NP (**Figure** **21**a). The GSDM‐A3 could efficiently contribute to the pore formation of the plasma membrane and release a large number of inflammatory factors. Despite merely 15% tumor cells being found to perform pyroptosis, the entire 4T‐1 tumor was completely eradicated in the cooperative treatment of Phe‐BF3 and GSDMA‐NP (Figure 21b). Nevertheless, this phenomenon did not appear in immune‐deficient mice, suggesting that abundant immune cells were involved in the tumor‐inhibitory process. The evident shrinkage of tumor volume and an obvious decrease of tumor weight were exhibited in in vivo experiments (Figure 21c). Furthermore, the noticeable increase of CD4^+^, CD8^+^, and NK cells infiltration within tumor also indicated the critical function of GSDM agonist for improving the antitumor immunotherapy (Figure 21d). Hence, these as‐synthesized GSDMA nanoconjugates provide the unique paradigm for the development of pyroptotic inducers based on GSDM protein family.

**Figure 22:**
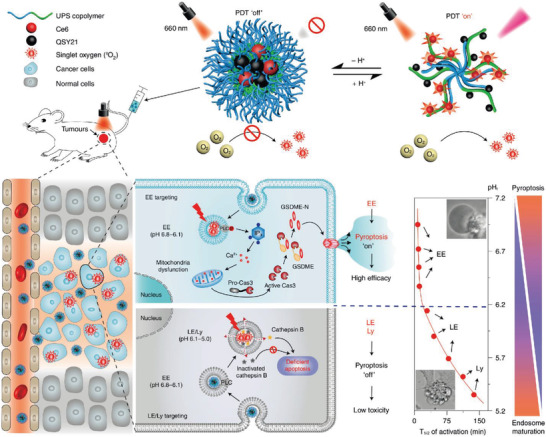
Schematic representation of ANPS and the fine‐tunable pyroptosis induced by acidity‐sensitive ANPS during the endosome maturation pathway. Reproduced with permission.^[^ ^69^ ^]^ Copyright 2022, Springer Nature.

Cellular endosomes and lysosomes play responsible roles in physiological activities, including cellular proliferation, lipid/protein catabolism, and even cell death.^[^ ^66^ ^]^ The pH values of endosomes commonly range from 5.5 to 6.8, and lysosomes have the optimal pH value of around 5.0.^[^ ^67^ ^]^ More attention has been paid to the fact that certain nanoparticles can be targeted into endosomes/lysosomes and induce various kinds of PCD, such as autophagy, ferroptosis, necroptosis, and pyroptosis.^[^ ^68^ ^]^ However, whether the entire endosomal/lysosomal maturation process is involved in the pyroptotic pathway and how to regulate the pyroptotic activity of bioactive nanomaterials remains largely unclear. Very recently, an acid‐activatable nanophotosensitizer (ANPS) library (based on mPEG‐bP(R1‐rR2)) was fabricated with a visible pH transition (pHt) from 5.3 to 6.9, covering the whole pH range of the endosomal maturation process (**Figure** **22**).^[^ ^69^ ^]^ The developed ultra‐pH‐sensitive nanoparticles could trigger an extensive GSDME‐mediated pyroptotic pathway through phospholipase C (PLC) activation caused by PDT‐induced oxidative stress in early endosomes (pH 6.8–6.1). In contrast, the pyroptotic activity was dramatically inhibited in late endosomes (pH 6.1–5.5) and lysosomes (pH 5.5–4.5), which was resulted from the PLC absence and cathepsin B inactivation in late endosomes and late lysosomes stages. GSDME is an essential substrate for pyroptosis execution. The ANPS‐library could efficiently induce maturation‐dependent GSDME‐FL cleavage into effective GSDME‐N fragments, which demonstrated the early endosome‐targeted ANPS could efficiently initiate a tunable pyroptosis through endosome maturation. Furthermore, genetic knockout research subsequently verified GSDME^−/−^ effectively avoided pyroptosis induction, switching cell pyroptosis into apoptosis in tumor cells. This study not only offers novel insights into cell pyroptosis by acidity‐responsive nanomaterials through endosomal maturation, but also provides an opportunity to exploit the pyroptosis‐mediated cancer immunotherapy.

**Figure 23:**
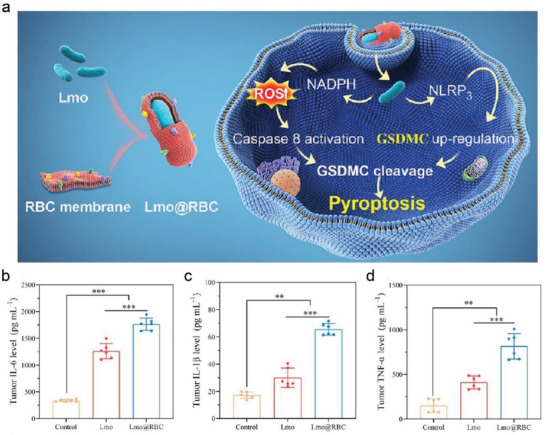
a) Schematic illustration of the synthetic procedure of *Lmo*@RBC, which were efficiently accumulated in primary tumor and then drove cancer cell pyroptosis caused by ROS generation. b–d) The level of IL‐6, IL‐1*β*, and TNF‐*α* in blood serum after different treatments. Reproduced with permission.^[^ ^72^ ^]^ Copyright 2022, American Chemical Society.

As one of the therapeutic bacteria, listeria monocytogenes (*Lmo*) can effectively accumulate and proliferate in malignant tumors with the help of tumor immunosuppressive microenvironment, but meanwhile, *Lmo* can also inhibit the development of tumor cells by generating excessive reactive oxygen species (ROS).^[^ ^70^ ^]^ In order to prevent the fast clearance and accompanied adverse effects, cell membrane coating has been proposed as an efficient biomimetic strategy that is helpful to solve the issues mentioned above.^[^ ^71^ ^]^ For instance, *Lmo* coated with red blood cell (RBC) membranes was constructed to drive cascade pyroptosis for antitumor immunotherapy without obvious biotoxicity.^[^ ^72^ ^]^ Profiting from the tumor‐homing properties of RBC membranes, *Lmo*@RBC could successfully accumulate in tumor sites. Subsequently, high‐enriched *Lmo*@RBC not only killed tumor cells through ROS production caused by NADPH oxidase, but also activated caspase‐8 to cleave GSDMC for pyroptosis induction (**Figure** **23**a). To elaborately elucidate the potential mechanism of *Lmo*@RBC, the transcriptome analysis of therapeutic tumor tissues was further conducted. The activation pathway of *Lmo*@RBC was closely correlated with the infiltration of immune cells. Compared with the control and *Lmo* treatments, the tumor‐bearing mice treated with *Lmo*@RBC displayed an obviously high level of IL‐6, IL‐1*β*, and TNF‐*α* in blood serum (Figure 23b–d), which was attributed to the high‐efficiency tumor‐homing capability of *Lmo*@RBC to improve the therapeutic efficiency. Triggered cell pyroptosis effectively promoted the DCs' maturation and activated antitumor immunity, thereby showing a highly suppressive effect on the proliferation and distant metastasis of malignant cancers. Therefore, the biomimetic strategy also enhanced the pyroptotic efficiency because of the excellent tumor targeting, low immunogenicity and desirable biocompatibility.

## 5 Conclusions and Perspectives

An emerging agreement is that complex molecular regulation of intrinsic signal pathway‐adjusted death procedures, commonly regarded as PCD, determines the malignant transformation and therapeutic potency of diseases. For a long time, apoptosis has been referred to a predominant mode of PCD underlying cancerous pathogenesis and therapeutic outcome. However, malignant tumors activate multiple oncogenic effectors by blocking the process of cell proliferation and resisting the tendency of cell apoptosis to survive.^[^ ^2^ ^]^ Hence, from a therapeutic perspective, there may be other opportunities for exploiting non‐apoptotic approaches to augment sensitivity and overcome resistance of therapies in malignant tumors. Pyroptosis, an emerging type of PCD, expands the traditional treatment concept and establishes its indispensable role in anti‐tumor therapy. Thanks to advanced nanomedicine, the cooperated application with nanoparticles can effectively compensate for current pyroptosis‐induced therapeutic limitations and defects. For instance, apart from the passive targeting based on the EPR effect and the active targeting after surface modification, the unique physicochemical properties and biological effects of nanoparticles can augment cancer‐pyroptotic efficacy and specificity, simultaneously minimizing harmful damage to normal organs/tissues. Despite the significant advances of nanomedicine‐augmented pyroptotic tumor nanotherapy, there are still several important and critical issues impeding the further possible clinical application of this therapeutic modality.

(1) **Design and manufacture of biocompatible pyroptotic nanoparticles**. Pyroptosis induction not only inhibits tumor proliferation but also exposes normal cells/tissues to the inflammatory environment for a long time. Compared with the traditional methods of small molecule‐inducing pyroptosis, nanotechnology provides an opportunity to effectively deliver the optimal dose of pyroptotic nanoparticles or therapeutic drugs into cancerous cells or tissues. Although some unique inorganic nanoparticles themselves can be employed as local sources for pyroptosis induction through external energy fields, the virulent metal ions produced from their degradation might also trigger pyroptotic toxicity in normal cells/tissues. Meanwhile, the organic nanoparticles also cause unsatisfactory accumulation within cancers due to the disadvantages of low stability and easy degradation in systemic circulation. The subsequent reach goal is how to tailor and engineer the nanomaterials with the desirable structural and compositional parameters in terms of size, shape, microstructure, and surface property for further enhancing the pyroptotic potency while weakening and mitigating the adverse effects. The fast and significant advance and progress in nanobiotechnology and nanomedicine is highly expected to find and supplement more desirable solutions to these critical issues.
(2) **Surface engineering of pyroptotic nanomaterials**. While pyroptosis participates in the immune defense against various malignant tumors, bacterial and viral infections, the dysregulation of pyroptosis induced by certain nanomaterials may lead to the low efficiency of pathogen clearance and dysfunction in the stimulation of adaptive immune defenses, giving rise to irreversible tissue damage.^[^ ^73^ ^]^ To achieve an excellent pyroptotic outcome with minimal side effects, these specific nanomaterials should be surface engineered to ensure the desirable tumor accumulation. Appropriate surface modification can preserve the long‐term systemic circulation and effectively avoid the phagocytosis of the reticuloendothelial system (RES). Of note, the surface‐targeted modification can realize active‐targeted accumulation into cancer cells, thereby improving the tumor pyroptosis potency. In addition, appropriate surface engineering can also significantly reduce the toxic effects of pyroptosis elicited by bioactive nanomaterials. The present surface‐engineering strategies of these pyroptotic nanomaterials are much less explored, which should be given more attention to in further research and study.
(3) **Optimizing the nanomedicine‐enabled/augmented pyroptotic antitumor efficiency**. The mechanism of traditional chemotherapy‐triggered pyroptosis has been thoroughly studied through a variety of biotechnologies. When co‐opted with nanotechnology into cancer therapy, the relevant pyroptotic processes and specific mechanisms may undergo significant changes due to the intricate in vivo tumor microenvironment. As an example, the critical executive protein GSDME is frequently silenced in cancer cells, most likely attributing to DNA promoter methylation,^[^ ^63^, ^74^ ^]^ and the presence of a tumor‐immunosuppressive microenvironment also downregulates or even suppresses the immunotherapeutic responses. In addition, the procedure of diverse nanomaterials to drive pyroptosis is not the same as each other. The associated mechanism research underlying the nanomedicine‐based pyroptotic therapy has not been fully revealed and understood, which brings difficulties to further optimizing the pyroptotic efficiency.
(4) **Broadening biomedical applications of nanomedicine‐enabled/augmented pyroptosis**. Pyroptosis initiated by bioactive nanomaterials has been extensively exploited in the majority of tumor treatments. It is highly anticipated that this kind of pyroptosis can be expanded to other biomedical fields involving in atherosclerosis therapy. Besides the synergistic therapy, the nanomedicine‐based pyroptotic therapeutics are also expected to be extensively integrated with other therapeutic modalities, which can further augment the therapeutic efficiency of pyroptosis‐based tumor therapy. Of note, except the mostly explored optical microscopic imaging techniques, there still lacks the efficient bioimaging modalities to directly and visibly characterize the occurrence of pyroptosis. It is also noted that certain pyroptotic nanomaterials also act as contrast agents in contrast‐enhanced diagnostic imaging patterns, thus it is highly possible and feasible to construct the imaging‐guided pyroptotic cancer therapy.
(5) **Biosafety and biocompatibility of pyroptosis nanoinducers**. The biosafety and biocompatibility of pyroptosis nanoinducers play a decisive role in speeding up their clinical translation. Despite the preliminary data having demonstrated the relatively excellent biosafety of certain pyroptosis nanoinducers, the long‐term toxicity and biological effects have not been fully exploited. Moreover, most of the pyroptosis nanoinducers are based on inorganic nanosystems, such as metal peroxide nanoparticles, which are still far away from the biosafety demonstration for next‐step clinical translation. More systematic in vitro and in vivo biocompatibility and biosafety assessments should be performed to offer evidence‐based results on biocompatibility and biosafety issues.

**Figure 24:**
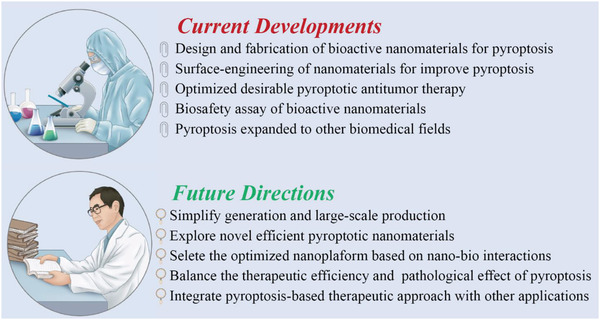
Summative scheme of the current development and future direction of nanomedicine‐enabled/augmented pyroptotic nanotherapy.

As one of the burgeoning tumor‐therapeutic approaches and interdisciplinary investigation field, nanomedicine‐enabled/augmented pyroptotic cancer therapy can amplify systemic inflammation to remodel the tumor microenvironment and improve satisfactory therapeutic efficiency for apoptosis‐escaping cancers. The roles of nanomedicine‐based pyroptosis in cancer research are still in the infancy stage, therefore further great efforts of the relevant signal pathways are required to be systematically investigated for benefiting personalized biomedicine in combating malignant diseases. It is strongly convinced that the cancer‐therapeutic modality involving pyroptosis will contribute meaningfully to the progress of nanomedicine and achieve promising clinical‐translation achievements to benefit an increasing number of cancerous patients, provided that these current critical issues and facing challenges are adequately addressed in future research (**Figure** **24**).

## Conflict of Interest

The authors declare no conflict of interest.
